# Comparison of baseline TEWL values in OFC positive and negative patients

**DOI:** 10.1111/pai.70376

**Published:** 2026-05-10

**Authors:** George E. Freigeh, Kelly M. O'Shea, Jonathan P. Troost, Bridgette Kaul, Christopher Launius, Lea M. Franco, Nicholas W. Lukacs, James R. Baker, Charles F. Schuler

**Affiliations:** ^1^ Division of Allergy and Clinical Immunology, Department of Internal Medicine Michigan Medicine Ann Arbor Michigan USA; ^2^ Mary H Weiser Food Allery Center, Michigan Medicine Ann Arbor Michigan USA; ^3^ Michigan Institute for Clinical & Health Research, Michigan Medicine Ann Arbor Michigan USA; ^4^ Department of Pathology Michigan Medicine Ann Arbor Michigan USA

**Keywords:** food allergy, medical device, oral food challenge, transepidermal water loss

**Abbreviations**
AD: atopic dermatitis
AUC: area under the curve
bTEWL: baseline transepidermal water loss
OFC: oral food challenge
ROC: receiver operator curve
TEWL: transepidermal water loss

To the Editor,

Food allergy is a pervasive chronic medical condition that affects up to 10% of adults and 8% of children.^1^ Despite such high disease prevalence, testing limitations often hinder accurate diagnosis and management of food allergy. Sensitization without confirmed food allergy for skin prick testing and serum IgE testing is reported up to 50%.^2^ The oral food challenge (OFC) therefore remains the gold standard of food allergy diagnosis. Unfortunately, the perceived risk of anaphylaxis remains a major barrier for more widespread OFC usage, despite the excellent safety profile of the procedure.^3^ There is thus a need for better food allergy anaphylaxis predictors to extend the potential reach and accuracy of food allergy testing and diagnosis. Transepidermal water loss (TEWL) is the normal occurrence of insensible water loss through the skin.^4^, ^5^ TEWL is measured as the water efflux from the skin surface in grams per square meter per hour (g/m^2^/h) with typical values ranging from 4 to 10 g/m^2^/h.^4^ TEWL is well established as a measure of skin barrier permeability, especially in the context of atopic dermatitis (AD) wherein TEWL is positively associated with AD even in non‐lesional skin.^6^ There is a clear association of epithelial barrier dysfunction with virtually all aspects of atopy, including food allergy.^7^ Identifying an operative, objective, and clinically accessible measure of barrier dysfunction is therefore a priority. Previous work from our group has demonstrated that TEWL rises dynamically during OFC reactions before the signs and symptoms of anaphylaxis.^4^ We sought to investigate whether baseline TEWL (bTEWL) prior to OFC could predict OFC outcomes.

All patients undergoing a clinical OFC as well as research OFC participants at our institution's food allergy clinics from June 2021 to December 2023 were eligible to participate in this study. All participants underwent an open label OFC in accordance with previously published guidelines.^8^ In this study, anaphylaxis was defined as acute onset of skin symptoms and at least one other organ system involvement or respiratory and/or cardiovascular compromise.^9^ Anaphylaxis severity was independently coded by study allergists who reviewed the documented clinical data using CoFAR criteria. TEWL was measured on the volar forearm in triplicate, and values were averaged to indicate the participant's baseline measurement while simultaneously excluding values with unacceptable variability. Active lesions were avoided. Measurements were recorded using the Tewameter Hex device (Courage + Khazaka). Further details on standardization of TEWL measurements and techniques are reported elsewhere.^4^ All analyses were performed with GraphPad Prism (GraphPad Software) and SAS (SAS Institute). We found that bTEWL distribution was asymmetrical (Figure S1) and so performed relevant analyses using non‐parametric tests. Informed consent was obtained from participants and this study was approved by our Institutional Review Board.

While we did record a limited dataset among those over 18 years old, that group included no OFC reactions; therefore, this analysis excluded participants over age 18 for a total of 208 participants. Demographic information is summarized in Table 1. OFC data, including wheal and serum IgE by food, are summarized in Table S1. When looking at participants of all ages, bTEWL was not significantly higher in reactors of any severity (*n* = 36, median = 13.02, IQR = 8.83–16.39) as compared to nonreactors (*n* = 172, median = 11.10, IQR = 9.31–13.53) (*p* = .16) or when comparing anaphylaxis (defined as CoFAR score greater than or equal to 2) (*n* = 19, median = 13.36, IQR = 8.88–17.27) to that of nonreactors (*p* = .10) (Figure 1A–C). We specifically evaluated the subgroup of participants age 5 and under given most reactions occurred in this age group. In participants age 5 and under, bTEWL was significantly higher among reactors (*n* = 20, median = 13.92, IQR = 10.52–18.98) as compared to nonreactors (*n* = 111, median = 11.30, IQR = 9.44–13.84) (*p* = .02). This effect was more pronounced when comparing anaphylaxis (*n* = 10, median = 16.30, IQR 11.95–22.57) to nonreactors (*p* = .009). There was a significant difference in mean bTEWL in correlation with CoFAR score in this age group, with a higher CoFAR score associated with a higher bTEWL (*p* = .01) (Figure 1D–F). There was no clear correlation between symptom type and bTEWL. We performed a subgroup analysis within a single food to test whether TEWL measurements might add to the usual predictive capacity of baseline testing. We selected peanut OFCs as these encompassed the largest number of reactions in a single food group (*n* = 13) in participants aged 5 and under. A logistic regression model using peanut wheal alone produced an AUC 0.6765, Ara h 2 specific sIgE alone produced an AUC of 0.8272, and bTEWL alone produced an AUC of 0.7206 (Figure 2A–C). To again account for influence of AD history, we performed a sensitivity analysis with the bTEWL model in participants age 5 and under. The point estimate was again relatively unchanged, with a point estimate of bTEWL alone of 1.167 and a point estimate of bTEWL with AD of 1.140 (Table S2). A regression model including only wheal and Ara h 2 specific sIgE produced an AUC of 0.7279 (Figure 2D). When bTEWL was added to the regression model with wheal, AUC increased to 0.8088 though when added to a regression model with Ara h 2 specific sIgE, AUC decreased to 0.7647 (Figure 2E,F). A combined regression model with all three parameters produced an AUC of 0.8088 (Figure 2G). A summary of point estimates and confidence intervals per model and effect is found in Table S3.

**TABLE 1:** Demographic information of participants.

	Overall	Age ≤ 5	Age ≥ 6
Age (year)			
*n*	208	131	77
Median (Q1–Q3)	4.51 (2.60–8.55)	2.90 (1.94–4.23)	11.40 (7.83–14.58)
Range	0.77–18	0.77–5	6–18
Mean (SD)	6.19 (4.78)	3.10 (1.39)	11.49 (3.78)
Sex, *n* (%)			
Male	133 (64%)	80 (61%)	53 (68%)
Female	76 (36%)	51 (39%)	25 (32%)
Race and Ethnicity, *n* (%)			
White	158 (76%)	104 (79%)	54 (70%)
Black	12 (6%)	8 (6%)	4 (5%)
Asian	27 (13%)	15 (11%)	12 (15%)
Hispanic	6 (3%)	3 (2%)	3 (4%)
Not specified	5 (2%)	1 (1%)	4 (5%)
History of AD			
Yes	146 (70%)	100 (76%)	46 (60%)
No	62 (30%)	31 (24%)	31 (40%)
OFC food, *n* (%)			
Peanut	42 (20%)	31 (24%)	11 (14%)
Egg	66 (32%)	49 (37%)	17 (22%)
Dairy	23 (11%)	13 (10%)	10 (13%)
Tree nut	54 (26%)	24 (18%)	30 (39%)
Other	23 (11%)	14 (11%)	9 (12%)
Reactors, *n* (%)	36 (17%)	20 (15%)	16 (21%)
Reactors with AD, *n* (%)	23 (64%)	16 (80%)	7 (44%)
CoFAR Scores in Reactors, *n* (%)			
1	17 (47%)	10 (50%)	7 (44%)
2	14 (39%)	7 (35%)	7 (44%)
3	5 (14%)	3 (15%)	2 (13%)
Baseline TEWL (g/m^2^/h)			
Mean (SD)	12.03 (4.27)	12.54 (4.40)	11.15 (3.90)
Median (Q1‐Q3)	11.21 (9.22–13.98)	11.50 (9.61–14.63)	10.84 (8.22–13.36)
Range	3.68–28.13	3.68–28.13	4.92–24.10

Abbreviations: AD, atopic dermatitis; SD, standard deviation; Q1–Q3, first and third quartile range.

**FIGURE 1:**
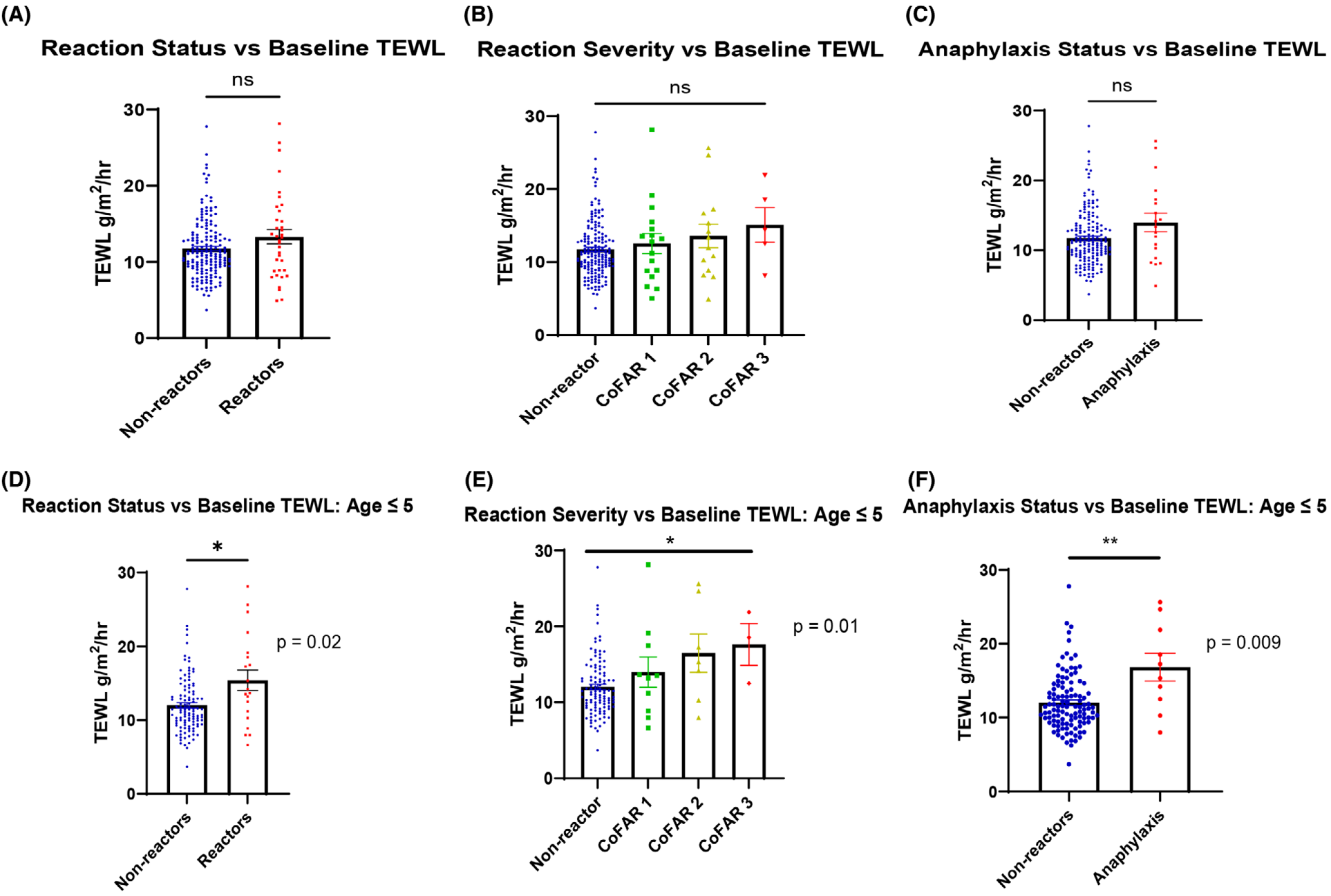
Reaction status and severity in association with baseline TEWL and atopic dermatitis. (A–C) indicate results for all participants; (D–F) indicate results for participants age 5 and under. Comparisons performed with Mann–Whitney test and Kruskal–Wallis test.

**FIGURE 2:**
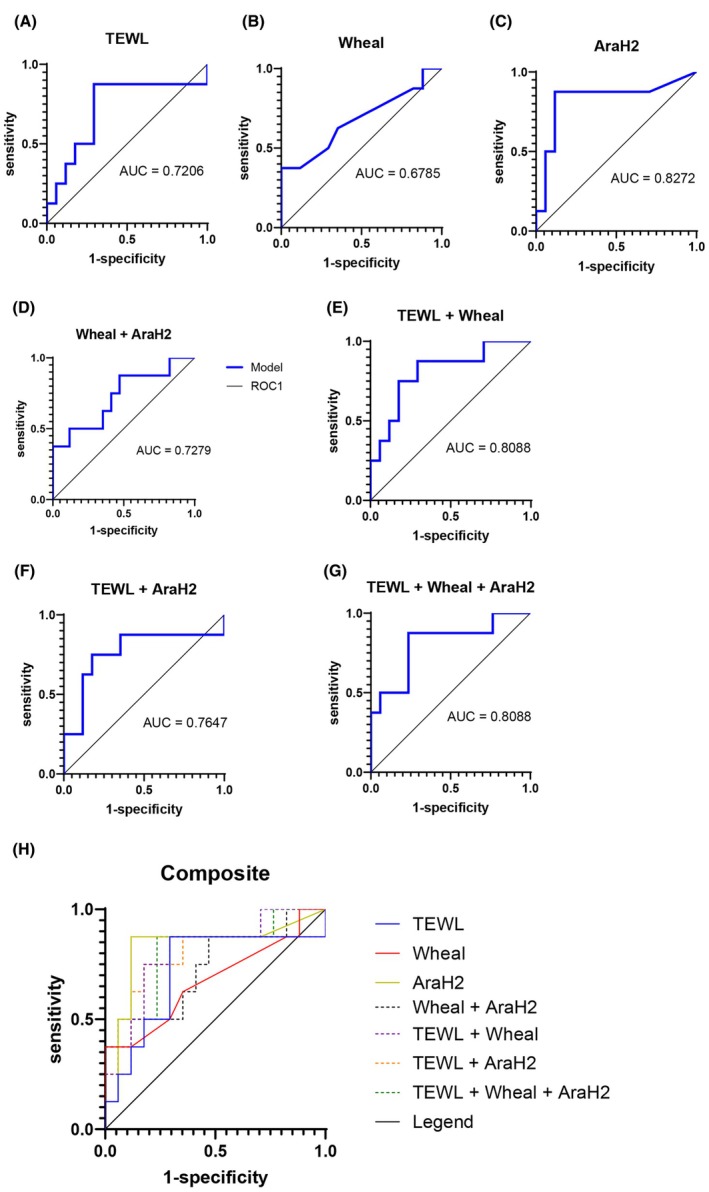
Receiver operator curves with TEWL, peanut wheal, and Ara h 2 specific serum IgE for peanut challenges in participants age 5 and younger. Reaction during OFC was considered a positive outcome.

Overall, these data support a positive correlation between bTEWL and OFC outcomes in young children. This is clinically relevant since anaphylaxis risk is an outcome often cited for OFC avoidance.^3^ The precise mechanism for the association of bTEWL with OFC outcome remains unclear, though prior work suggests a mechanistic connection between persistent epithelial barrier dysfunction and food allergy.^10^ TEWL may therefore serve as a surrogate measurement of such barrier dysfunction. In our subgroup analysis of peanut OFCs, bTEWL helped improve the predictive accuracy of peanut wheal and Ara h 2 specific sIgE models, again supporting bTEWL as a contributory marker in assessing OFC risk. However, it should be noted that due to the small number of reactors, it is difficult to implement a robust multivariable regression with multiple variables. Additionally, in this current study, we cannot fully separate the contribution of AD to its effect on bTEWL and to what degree bTEWL serves as a completely independent variable. It is increasingly clear that epithelial function plays a prominent role in atopic disorders' development and progression and that variants in epidermal genes such as those encoding filaggrin and SPINK5 have been found to confer food allergy risk independent of AD.^11^, ^12^ Integrating TEWL into future genetic profiling studies could yield interesting results in how these variants lead to direct barrier dysfunction that can be measured in vivo in a clinical setting. This study has limitations. Future work would benefit from standardized challenge contexts, such as a high or low reaction likelihood food challenge trial to verify whether bTEWL correlates with reaction status in one context or the other. Additionally, this study has not investigated mechanisms for the association of TEWL with food allergy; our research group is actively pursuing the mechanistic considerations of this phenomenon to address this limitation in future work. These data provide further evidence of a link between skin barrier dysfunction and food allergy, particularly in young children. TEWL may serve as an additional patient diagnostic marker utilized in assessing OFC risk in addition to usual testing. Ultimately, we expect that better risk prediction will contribute to making OFCs more accessible and acceptable to both patients and providers.

## AUTHOR CONTRIBUTIONS

**George E. Freigeh:** Conceptualization; investigation; writing – original draft; writing – review and editing; visualization; formal analysis. **Kelly M. O'Shea:** Conceptualization; investigation; writing – review and editing. **Jonathan P. Troost:** Writing – review and editing; formal analysis; data curation; visualization. **Bridgette Kaul:** Investigation; project administration. **Christopher Launius:** Investigation. **Lea M. Franco:** Investigation; writing – review and editing; project administration. **Nicholas W. Lukacs:** Conceptualization; supervision; resources. **James R. Baker Jr:** Conceptualization; supervision; resources. **Charles F. Schuler IV:** Conceptualization; investigation; funding acquisition; writing – review and editing; resources; supervision; formal analysis; methodology.

## FUNDING INFORMATION

The funders had no role in the study design, data collection and interpretation, or the decision to submit the work for publication. CS received support from the University of Michigan via a Mary H. Weiser Food Allergy Center Michigan Food Allergy Accelerator (MFARA) pilot grant and the Ronald Koenig, MD, PhD Department of Internal Medicine Early Career Endowment; the Gerber Foundation (award number 9026); and the National Institute of Allergy and Infectious NLDiseases (NIAID) of the NIH (award number K23AI162661). JT receives support from the NIH (award number UM1TR004404). NL receives support from Food Allergy Research and Education via a Discovery Center of Excellence award. The content is solely the responsibility of the authors and does not necessarily represent the official views of the NIH. Analytic support was provided in part by NUCATS: UM1TR004404.

## CONFLICT OF INTEREST STATEMENT

GEF, KO, JT, BK, CL, and LF have declared no conflict of interest exists. CFS, NL, and JB have submitted a patent application (US Patent Application no. 18/730391) involving transepidermal water loss measurement in predicting anaphylaxis.

## Supporting information


Appendix S1.

## Data Availability

The data that support the findings of this study are available on request from the corresponding author. The data are not publicly available due to privacy or ethical restrictions.
