# Supplementary material for: Comparison of baseline TEWL values in OFC positive and negative patients
Source: Pediatr Allergy Immunol. 2026 May 10;37:e70376. doi: 10.1111/pai.70376 (PMC13157688; doi:10.1111/pai.70376)
Supplement: Supplementary file 1 — Appendix S1. [file PAI-37-e70376-s001.docx]

**Supplemental Figure 1:** Distribution of baseline TEWL values in all participants demonstrating a non-normal distribution.


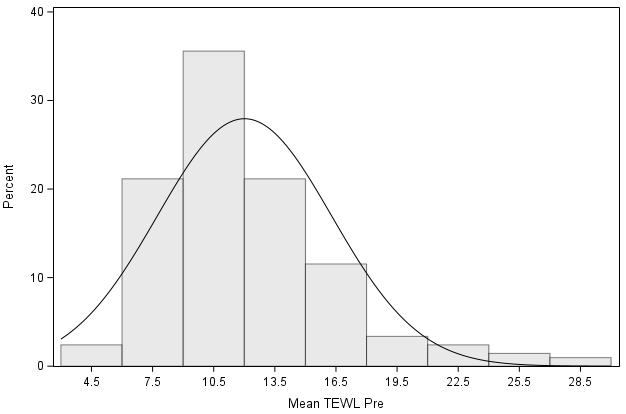


Percent

Baseline TEWL

**Supplemental Table 1:** Food-specific challenge and testing information. The tree nuts are grouped together, though testing data represented here is specific to the nut challenged. Abbreviations: Q1-Q3, first and third quartile range; SD, standard deviation.

|  | Overall | Age ≤ 5 | Age ≥ 6 |
| --- | --- | --- | --- |
| Peanut Challenges |  |  |  |
| Total, n | 42 | 31 | 11 |
| Reactors, n (%) | 18 (40%) | 13 (72%) | 5 (28%) |
| Peanut SPT Wheal (mm) Median (Q1-Q3) | 6.00 (4-8.25) | 5.00 (4-7) | 6.00 (4-10) |
| Total Peanut Serum IgE (kU/L) Median (Q1-Q3) | 0.83 (0.09-2.41) | 0.54 (0-1.26) | 1.21 (0.54-4.18) |
| Peanut AraH2 Serum IgE (kU/L) Median (Q1-Q3) | 0.25 (0.07-0.85) | 0.25 (0.1-0.53) | 0.43 (0.04-1.25) |
| Egg Challenges |  |  |  |
| Total, n | 66 | 49 (74%) | 17 (26%) |
| Reactors, n (%) | 7 (11%) | 5 (71%) | 2 (29%) |
| Egg SPT Wheal (mm) Median (Q1-Q3) | 4.00 (3-7.25) | 4.00 (3-7) | 5.00 (4-8) |
| Egg White Serum IgE (kU/L) Median (Q1-Q3) | 1.47 (0.19-4.27) | 1.13 (0-3.65) | 2.42 (1.1-5) |
| Cow's Milk Challenges |  |  |  |
| Total, n | 23 | 10 (43%) | 13 (57%) |
| Reactors, n (%) | 2 (9%) | 1 (50%) | 1 (50%) |
| Cow's Milk SPT Wheal (mm) Median (Q1-Q3) | 7.00 (2-10) | 7.00 (2-9.5) | 8.00 (6-10) |
| Cow's Milk Serum IgE (kU/L) Median (Q1-Q3) | 1.78 (0.44-4.01) | 2.22 (0.72-4.91) | 0.69 (0.10-1.49) |
| Tree Nut Challenges |  |  |  |
| Total, n | 54 | 24 (44%) | 30 (56%) |
| Reactors, n (%) | 7 (13%) | 1 (14%) | 6 (86%) |
| Specific Tree Nut SPT Wheal (mm) Median (Q1-Q3) | 3.00 (1-5) | 1.00 (0-3) | 4.00 (2-6.5) |
| Specific Total Tree Nut Serum IgE (kU/L) Median (Q1-Q3) | 0.35 (0-1.46) | 0.47 (0-0.78) | 0 (0-1.84) |

**Supplemental Table 2:** Summary of point estimates and confidence intervals of TEWL with and without AD for all peanut challenges. Cells marked “NA” are due to the fact that there are no non-AD reactors in the age 5 and under group and so the estimate approaches infinity

| **Model (all ages)** | **Odds Ratio Estimates** | | | |
| --- | --- | --- | --- | --- |
|  | **Effect** | **Point Estimate** | **95% Wald Confidence Limits** | |
| TEWL | TEWL | 1.134 | 0.975 | 1.318 |
| TEWL + AD | TEWL | 1.123 | 0.961 | 1.313 |
|  | AD | 1.484 | 0.231 | 9.534 |
| **Model (age 5 and under)** | **Odds Ratio Estimates** | | | |
|  | **Effect** | **Point Estimate** | **95% Wald Confidence Limits** | |
| TEWL | TEWL | 1.167 | 0.978 | 1.392 |
| TEWL + AD | TEWL | 1.140 | 0.950 | 1.366 |
|  | AD | N/A | N/A | N/A |

**Supplemental Table 3**: Summary of point estimates, confidence intervals, and p values for receiver operator curves (ROC) per model for age 5 and under for peanut challenges. Abbreviations: AUC, area under the curve.

| **Model (age 5 and under)** |  | **Odds Ratio Estimates** | | | |  |
| --- | --- | --- | --- | --- | --- | --- |
|  | **AUC** | **Effect** | **Odd Ratio** | **95% Wald Confidence Limits** | | **p value** |
| TEWL | 0.7206 | TEWL | 1.167 | 0.978 | 1.392 | 0.0866 |
| Wheal | 0.6765 | Wheal | 1.283 | 0.962 | 1.712 | 0.0897 |
| AraH2 | 0.8272 | AraH2 | 2.212 | 0.679 | 7.201 | 0.1875 |
| Wheal + AraH2 | 0.7279 | Wheal | 1.220 | 0.875 | 1.700 | 0.2417 |
|  |  | AraH2 | 1.786 | 0.501 | 6.367 | 0.3715 |
| TEWL + Wheal | 0.8088 | TEWL | 1.168 | 0.971 | 1.407 | 0.1002 |
|  |  | Wheal | 1.324 | 0.940 | 1.863 | 0.1078 |
| TEWL + AraH2 | 0.7647 | TEWL | 1.150 | 0.951 | 1.389 | 0.1492 |
|  |  | AraH2 | 2.246 | 0.641 | 7.873 | 0.2062 |
| TEWL + Wheal + AraH2 | 0.8088 | TEWL | 1.174 | 0.967 | 1.426 | 0.1052 |
|  |  | Wheal | 1.284 | 0.893 | 1.848 | 0.1779 |
|  |  | AraH2 | 1.916 | 0.502 | 7.313 | 0.3412 |
